# Percutaneous Coronary Intervention Complexity and Risk of Adverse Events in relation to High Bleeding Risk among Patients Receiving Drug-Eluting Stents: Insights from a Large Single-Center Cohort Study

**DOI:** 10.1155/2020/2985435

**Published:** 2020-04-25

**Authors:** Hao-Yu Wang, Yang Wang, Dong Yin, Run-Lin Gao, Yue-Jin Yang, Bo Xu, Ke-Fei Dou

**Affiliations:** ^1^Department of Cardiology, Coronary Heart Disease Center, Fuwai Hospital, National Center for Cardiovascular Diseases, Chinese Academy of Medical Sciences and Peking Union Medical College, Beijing 100037, China; ^2^Medical Research & Biometrics Center, National Center for Cardiovascular Diseases, Fuwai Hospital, Chinese Academy of Medical Sciences and Peking Union Medical College, Beijing 100037, China; ^3^Department of Cardiac Catheterization Laboratories, Fuwai Hospital, National Center for Cardiovascular Diseases, Chinese Academy of Medical Sciences and Peking Union Medical College, Beijing 100037, China

## Abstract

**Background/Aim:**

The relation between complex percutaneous coronary intervention (PCI), high bleeding risk (HBR), and adverse events after coronary artery implantation of drug-eluting stents has been incompletely characterized. This study sought to investigate the ischemic and bleeding events after complex PCI including stratification according to HBR estimated by PARIS bleeding risk score.

**Methods:**

Between January 2013 and December 2013, 10,167 consecutive patients undergoing PCI were prospectively enrolled in Fuwai PCI Registry. Complex PCI was defined when having at least one of the following characteristics: 3 vessels treated, ≥3 stents implanted, ≥3 lesions treated, bifurcation with 2 stents implanted, total stent length >60 mm, treatment of chronic total occlusion, unprotected left main PCI, in-stent restenosis target lesion, and severely calcified lesion. The primary ischemic endpoint was major adverse cardiovascular events (MACE) (composite of cardiac death, myocardial infarction, definite/probable stent thrombosis, and target lesion revascularization), and primary bleeding endpoint was Bleeding Academic Research Consortium (BARC) type 2, 3, or 5 bleeding.

**Results:**

The median duration of follow-up was 29 months. In adjusted Cox regression analysis, patients having complex PCI procedures experienced higher risks of MACE (hazard ratio (HR): 1.63, 95% confidence interval (CI): 1.38–1.92; *P* < 0.001), compared with noncomplex PCI. In contrast, the risk of clinically relevant bleeding was statistically similar between the 2 groups (HR: 0.86 [0.66–1.11]; *P* = 0.238). There was no statistical interaction between HBR (PARIS bleeding score ≥8 or <8) and complex PCI in regard to MACE (adjusted *P*_interaction_ = 0.388) and clinically relevant bleeding (adjusted *P*_interaction_ = 0.279).

**Conclusions:**

Patients who had undergone complex PCI resulted in substantially more ischemic events, without an increase in clinically relevant bleeding risk, and these associations did not seem to be modified by HBR status. More intensified antiplatelet therapy may be beneficial for patients with complex percutaneous coronary revascularization procedures.

## 1. Introduction

Due to advances in intervention techniques and technologies [1, 2], percutaneous coronary interventions (PCIs) are increasingly performed in complex clinical and anatomical subsets of patients although literature data showed steady declines in population-wide rates of coronary revascularization over the past decade [3]. Given the association of anatomical complexity and functional severity of CAD with future cardiovascular events [4, 5], the concept of complex PCI and higher-risk indicated population for revascularization has recently been proposed [6, 7]. However, there is no universal definition of complex PCI in terms of angiographic and lesion characteristics, in turn causing in a variety of clinical outcomes reported in previous studies [7–12]. Although procedural complexity emerges as a correlate of ischemic events, controversial results have been reported in many studies evaluating the adverse impact of complex PCI procedures on bleeding events [7–12]. For instance, some reports suggested the increased risk of bleeding events in patients with high-risk features for stent-related ischemic events [10–12], whereas others refuted this association [7–9, 13]. Meanwhile, concomitant high bleeding risk (HBR) may be present, making it challenging for clinical decision-making on the duration and intensity of dual antiplatelet therapy (DAPT) after complex PCI. Currently, limited data are available regarding the effect of complex percutaneous coronary revascularization procedures on clinical outcomes in a real-world population, especially in East Asian Patients.

In clinical practice, because patients with high bleeding risk (HBR) who undergo PCI experience both high rates of ischemic and bleeding events and represent an overall high-risk population [14, 15], whether complex PCI procedures exert similar or differential impact on both thrombotic and bleeding complications among those with and without HBR is uncertain. Hence, determining the optimal strategy for DAPT in patients after complex PCI procedures requires the individualized assessment of the patient's risk of ischemia and bleeding. To date, the PARIS bleeding risk score is a 6-item scoring system developed to estimate the bleeding risk in patients who receive DAPT after drug-eluting stent (DES) implantation [16], which drives its endorsement by 2016 ACC/AHA DAPT guidelines [17]. In the derivation and validation study of PARIS score [16, 18], the absolute risk difference in coronary thrombosis and major bleeding with prolonged dual antiplatelet therapy was largely negative for patients with high PARIS bleeding risk score, particularly for those at low or intermediate thrombotic risk. Specifically, it is known that complex PCI and HBR are intertwined and unfavorably affect prognosis after PCI, but the impact of HBR estimated by PARIS bleeding risk score on the occurrence of ischemic and bleeding events in the setting of complex PCI procedures with DES implantation is not well established.

Accordingly, we sought to (1) describe the ischemic and bleeding events of patients who underwent complex PCI procedures compared with noncomplex PCI and (2) examine whether HBR, as defined by the PARIS bleeding risk score, affects the association between procedural complexity and clinical outcomes differently in an unselected real-world population receiving PCI with DES.

## 2. Methods

### 2.1. Patient Population

This was a retrospective analysis of prospectively collected data. Between January 2013 and December 2013, a total of 10,724 consecutive patients who underwent PCI for CAD were prospectively enrolled from Fuwai Hospital, National Center for Cardiovascular Diseases, Beijing, China. For the present study, exclusion criteria were treatment by balloon angioplasty alone without stent placement, implantation of bioresorbable scaffolds, or bare-metal stents. Finally, 10,167 patients were selected for this analysis. Demographic and clinical characteristics, angiographic and procedural information, and follow-up data were systematically and prospectively collected in our dedicated PCI registry by independent research personnel. The study was conducted based on the principles of the Declaration of Helsinki, and its protocol was approved by the Institutional Review Board. All patients provided written informed consent for prospective follow-up before the intervention. Details of the clinical and laboratory analysis and procedures are contained in the Supplementary material method.

### 2.2. Patient Follow-Up

After index PCI, patients were followed up at 1, 6, and 12 months and annually thereafter. Follow-up data were collected through medical records, telephone communications, or clinical visits by well-trained cardiologists who were blind to the purpose of the present study. Patients were advised to return for coronary angiography if indications of ischemic events occurred. The median follow-up duration was 881 days (interquartile range (IQR): 807 to 944 days).

### 2.3. Definitions and Clinical Outcomes

Complex PCI was quantified with at least 1 element of the following characteristics: 3 coronary vessels treated, ≥3 stents implanted, ≥3 lesions treated, bifurcation with 2 stents implanted, total stent length >60 mm, treatment of chronic total occlusion (CTO), unprotected left main PCI, in-stent restenosis target lesion, and severely calcified lesion (requiring a rotablator system). The definition of complex PCI is an extended version of that proposed by Giustino et al. [7] and used in the ESC DAPT guidelines [19] and includes PCI for unprotected left main, in-stent restenosis, and heavily calcified lesion (using rotablation). Notably, these high-risk features are well recognized to predispose to higher rates of thrombotic events [20–22], but they were the exclusion criteria in a retrospective analysis using the pooled patient-level data of 6 randomized controlled trials [7]. Validation of the PARIS bleeding score and instruction for its calculation were described elsewhere [16, 18, 23]. Patients were deemed at HBR for scores ≥8 and non-HBR for scores <8.

The primary ischemic outcome was MACE, defined as a composite of cardiac death, MI, definite or probable ST, and target lesion revascularization (TLR). The primary bleeding outcome was clinically relevant bleeding defined as the Bleeding Academic Research Consortium (BARC) type 2, 3, or 5 [24]. Secondary outcomes were all-cause death, cardiac death, MI, TV-MI, definite/probable ST, any repeat revascularization, target vessel revascularization (TVR), TLR, stroke, and any bleeding. Detailed information on endpoint definitions is presented in the Supplementary material method.

### 2.4. Statistical Analysis

Continuous variables are expressed as mean ± SD or median (interquartile range) and compared with Student's *t*-test or the Mann–Whiney *U* test, respectively. Categorical data are reported as numbers and percentages and were compared using chi-square or Fisher's exact test as appropriate. Cumulative event rates for ischemic and bleeding events were constructed using Kaplan–Meier method among those with and without PCI complexity and after substratifying all subjects by both PCI complexity and HBR. Event rates were compared across groups using the log-rank test. Hazard ratios (HRs) with 95% confidence intervals (CIs) were calculated using a Cox proportional hazard regression model. A multivariable Cox regression model was used to compare the risks of adverse cardiac events between the complex PCI and noncomplex PCI groups using the following covariates: age, sex, current smoking, body mass index, hypertension, diabetes mellitus, chronic kidney disease, left ventricular ejection fraction, prior MI, prior revascularization (percutaneous coronary intervention and/or coronary artery bypass graft), acute coronary syndrome, mean stent diameter, hemoglobin, platelet count, type of DES implanted, and DAPT duration (as a time-adjusted covariate). “Complex PCI” was also assessed as either a categorical (0, 1 to 2, and ≥3) or a continuous (per increase in the number of complex PCI features) covariate in the Cox model. In addition, each complex PCI procedure component was included as a separate predictor in the multivariable Cox regression analysis to calculate individual predicted probabilities for MACE and clinically relevant bleeding. The consistency of the effect of undergoing complex PCI procedures according to HBR (HBR vs. non-HBR) was evaluated by formal interaction testing. Exploratory sensitivity analyses were performed to evaluate the consistency of our overall findings, including using three bleeding risk categories (low risk: 0 to 3, moderate risk: 4 to 7, and high risk: ≥8 points) of PARIS bleeding risk score and defining HBR according to PRECISE-DAPT score (i.e., ≥25). All tests were two-sided, and a *P* value of <0.05 was considered to be statistically significant. All analyses were performed with SAS version 9.4 (SAS Institute, Cary, NC, USA).

## 3. Results

### 3.1. Clinical and Procedural Characteristics

Of 10167 patients (mean age: 58.3 ± 10.3 years) with available angiographic characteristics, 3651 (35.9%) underwent complex PCI. The baseline and procedural characteristics according to PCI complexity are presented in Table 1. Patients who underwent complex PCI were more likely to be elderly and male with a high prevalence of diabetes mellitus and hypertension. The complex PCI group had a higher proportion of stable CAD as an indication for PCI, previous MI, and myocardial revascularization with either PCI or CABG. There were higher PARIS thrombotic risk scores in the complex PCI group without difference in PARIS bleeding risk score levels. Procedurally, the complex PCI group had a greater number of treated vessels and lesions with more numbers of stents implanted, leading to a greater total stent length. Subjects with complex PCI were more likely to display involvement of thrombotic lesion, type B2/C lesion, and higher SYNTAX scores. The prevalence of the complex PCI components in the overall population is illustrated in Supplementary Figure 1, and the overlap of these high-risk features is summarized in Supplementary Table 1. As expected, ≥ 3 stents implanted and ≥3 lesions treated frequently overlapped with other high-risk procedural characteristics.

### 3.2. Clinical Outcomes in relation to Complex PCI Procedures

**Figure 1:**
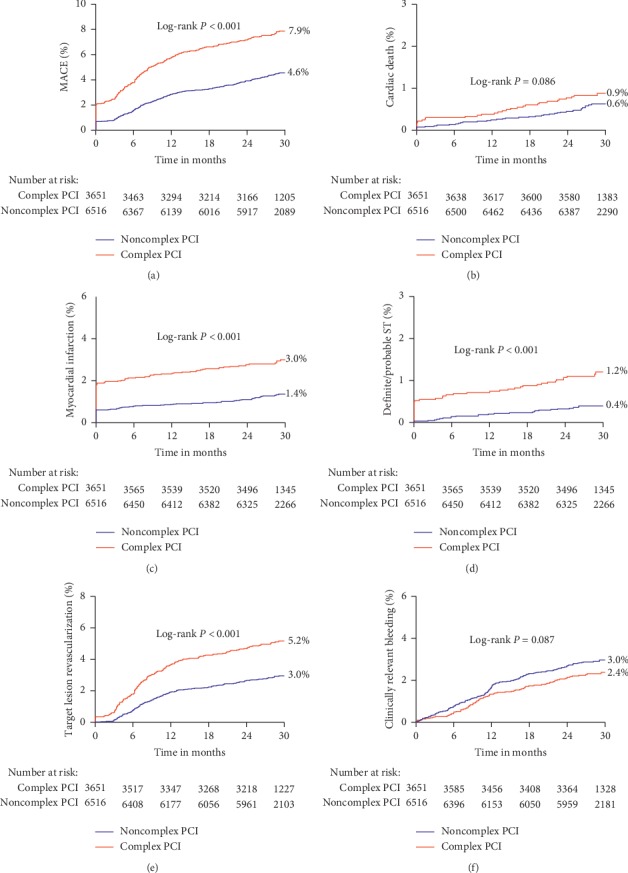
Kaplan–Meier cumulative event curves for (a) major adverse cardiovascular events (MACE), (b) cardiac death, (c) myocardial infarction (MI), (d) definite/probable stent thrombosis (ST), (e) target lesion revascularization (TLR), and (f) clinically relevant bleeding according to PCI complexity.

In crude analyses (Figure 1), patients who had complex PCI had higher Kaplan–Meier rates of the MACE (7.9% vs. 4.6%), MI (3.0% vs. 1.4%), definite/probable ST (1.2% vs. 0.4%), and TLR (5.2% vs. 3.0%; *P* < 0.001 for all). Nevertheless, there was no difference between patients with complex and noncomplex PCI in the rates of clinically relevant bleeding (2.4% vs. 3.0%; *P*=0.087). After multivariable adjustment, differences remained significant for MACE (adjusted HR: 1.63, 95% CI: 1.38–1.92; *P* < 0.001), MI (adjusted HR: 2.16 (1.62–2.87); *P* < 0.001), definite/probable ST (adjusted HR: 2.71 (1.66–4.41); *P* < 0.001), and TLR (adjusted HR: 1.59 (1.29–1.95); *P* < 0.001), whereas the adjusted risk of clinically relevant bleeding remained similar in both groups (adjusted HR: 0.86 (0.66–1.11); *P*=0.238) (Table 2).

By including complex PCI as a continuous variable within the same multivariable models, the risk of MACE tended to be greater as the number of high-risk procedural characteristics increased (per number of complex PCI variables increase, adjusted HR: 1.16, 95% CI: 1.09–1.23; *P* < 0.001) (Supplementary Table 2). Of note, the complex PCI score was not associated with greater risk of clinically relevant bleeding (adjusted HR: 0.91, 95% CI: 0.82–1.02; *P*=0.107). Besides, the number of PCI complexity was associated with greater risk of the primary ischemic endpoint. Conversely, there was a numerically gradual risk decrease for clinically relevant bleeding (0 : 2.9%; 1 to 2 : 2.4%; ≥3 : 2.2%; *P*=0.223) as the number of high-risk features increased. Adjusted risk for MACE and clinically relevant bleeding according to each component of high-risk procedural feature is illustrated in Supplementary Table 3. Individual high-risk features, such as ≥3 stents implanted, bifurcation with 2 stents, >60 mm total stent length, in-stent restenosis target lesion, and severely calcified lesion, are independent predictors for MACE but not for clinically relevant bleeding.

### 3.3. Clinical Outcomes in relation to Complex PCI Procedures and HBR

**Figure 2:**
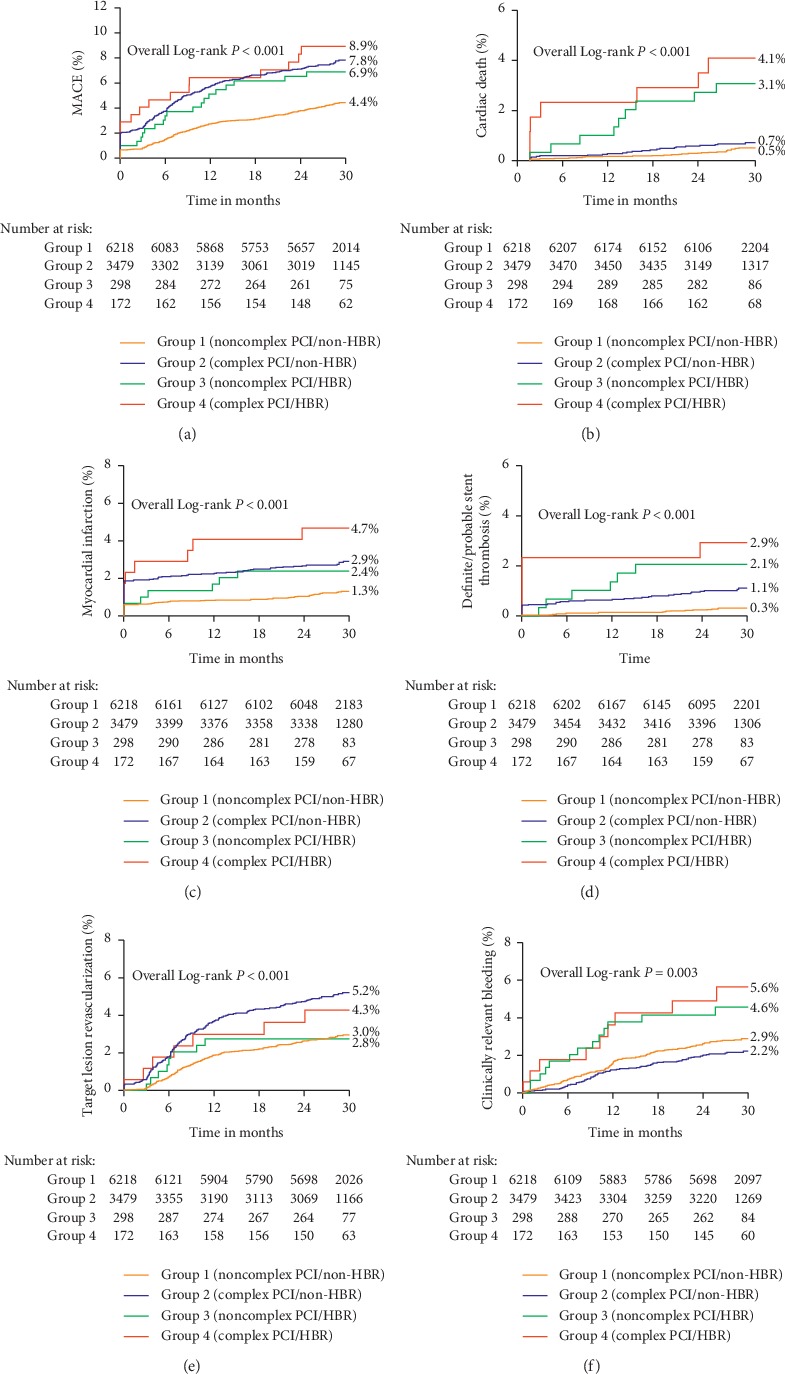
Kaplan–Meier cumulative event curves for (a) major adverse cardiovascular events (MACE), (b) cardiac death, (c) myocardial infarction (MI), (d) definite/probable stent thrombosis (ST), (e) target lesion revascularization (TLR), and (f) clinically relevant bleeding according to PCI complexity and HBR status.

Indeed, subjects with HBR had significantly greater rates of ischemic and bleeding events compared with subjects without HBR (Supplementary Table 4). As shown in Figure 2, the rates of MACE among subjects with both HBR and complex PCI, HBR alone, complex PCI alone, or neither HBR and complex PCI were 8.9%, 6.9%, 7.8%, and 4.4%, respectively (*P* < 0.001). Similar patterns of higher risk were observed for cardiac death, MI, or definite/probable ST. The rate of clinically relevant bleeding was higher among subjects with HBR, although complex PCI showed nonstatistically significant low rates of major bleeding (*P*=0.003). Clinically relevant bleeding rates across these same 4 groups were 5.6%, 4.6%, 2.2%, and 2.9%, respectively.

Adjusted HRs for ischemic and bleeding events associated with complex PCI procedures and stratified by the presence or absence of HBR are shown in Table 3. The HRs of any endpoint were similar in the direction and magnitude among the HBR and non-HBR groups with no evidence of statistical interaction (all *P*_interaction_ > 0.05), suggesting a consistent effect within complex PCI. There was no significant interaction (*P*=0.388) in the adverse effect of complex versus noncomplex PCI for MACE between patients with HBR (adjusted HR: 1.13, 95% CI: 0.57–2.25) and non-HBR (adjusted HR: 1.66, 95% CI: 1.40–1.97). The unadjusted rates of cardiac death, MI, definite/probable ST, and TLR were higher in HBR subjects with complex PCI in relation to HBR subjects without complex PCI; however, after multivariable adjustment, the HRs were not significantly different as analysis of subjects with HBR is limited by small sizes. It was worthy of noting that the risk of clinically relevant bleeding associated with complex PCI was not increased in participants with HBR (5.8% versus 4.4%; adjusted HR: 1.22, 95% CI: 0.52–2.85) and in those without HBR (2.2% versus 2.9%; adjusted HR: 0.82, 95% CI: 0.62–1.07; *P*_interaction_=0.269). Results were consistent when considering the three risk strata (low, intermediate, or high) bleeding risk in the light of PARIS bleeding risk score (Supplementary Table 5). Additionally, similar results were obtained when applying PRECISE-DAPT score to define HBR (PRECISE-DAPT score ≥25) (Supplementary Table 6). The ischemic and bleeding endpoints did not differ significantly in relation to complex PCI in sensitivity analyses stratified for HBR versus non-HBR group (all *P*_interaction_ > 0.05) except for MI (*P*_interaction_=0.031). Furthermore, the effect of complex versus noncomplex PCI on MACE (adjusted HR: 1.70 (1.32–2.18) with stable CAD and adjusted HR: 1.58 (1.26–1.97) with ACS, *P*_interaction_=0.575) and clinically relevant bleeding (adjusted HR: 1.01 (0.69–1.48) with stable CAD and adjusted HR: 0.74 (0.52–1.06) with ACS, *P*_interaction_=0.223) was similar regardless of the patient presented with stable CAD or ACS.

## 4. Discussion

The present study of more than 10,000 real-world patients undergoing PCI predominantly with new-generation DES is the first study to address the association between complex PCI procedures, HBR, and occurrence of adverse events in a large all-comers PCI cohort. The main findings of this analysis could be summarized as follows:

1. Compared with noncomplex PCI, PCI complexity was associated with a considerably higher risk of adverse ischemic events, with no higher risk of clinically relevant bleeding in multivariable analyses over median 29 months of follow-up. The ischemic risk tended to be greater for progressively higher degrees of procedural complexity.
2. The independent impact of complex PCI on thrombotic events was substantial and uniform irrespective of HBR status, and there was no interaction between complex PCI and HBR (i.e., PARIS bleeding score ≥8) on clinically relevant bleeding.
3. Together these findings indicated that regardless of HBR, complex PCI was an independent driver of adverse ischemic outcomes without an excess of bleeding events, suggesting that the use of potent P2Y_12_ inhibitors may be beneficial to patients who underwent complex percutaneous revascularization.

We observed that PCI complexity exerted an adverse impact not only on MACE proportional to the number of complexity criteria present, but also on all individual endpoints including cardiac death, MI, definite/probable ST, and TLR, findings that corroborated results from previous reports [7–12]. Intriguingly, subjects with complex PCI did not experience a significant increased risk of clinically relevant bleeding, as compared with the noncomplex PCI group. In this regard, three analyses from DAPT study, PROMETHEUS study, and a pooled patient-level data from six RCTs showing comparable clinically relevant bleeding risks between complex and noncomplex PCI groups were consistent with our findings [7–9, 13]. In contrast to these observations, the results of ADAPT-DES registry and Global Leaders trial showed that complex PCI criteria were correlated with a higher incidence of bleeding [10, 12]. Analogously, in the Bern PCI Registry consisting of 10,236 post-PCI patients, Ueki et al. also demonstrated a significant relationship of high-risk features for stent-related ischemic events with BARC 3–5 bleeding [11].

These conflicting results in regard to the impact of complex PCI on bleeding events may be attributable to differences in definition of “complex PCI,” intensity of DAPT (clopidogrel vs. more potent P2Y_12_ inhibitors), and the bleeding risk of the study population. Specifically, our own definition of complex PCI was an extended version of that proposed by Giustino et al. and included unprotected left main PCI, in-stent restenosis target lesions, and rotational atherectomy use for a heavy calcified lesion. Given that these high-risk features were established risk factors of thrombotic complications [20–22], such patients that were excluded from a patient-level pooled dataset from six RCTs are necessary to be taken into account in a real-world practice. Under this scenario, it is unsurprising that the proportion of patients receiving complex procedures was markedly higher (35.9%) in our study than in two previous pooled patient-level databases from RCTs that ranged from 17.9% to 29.6% [7, 12, 13]. Furthermore, high-risk features from the Bern PCI Registry were mainly comprised of CKD (47.6%) [11], which has emerged as a common contributor to both types of ischemic and bleeding complications [16, 25]. They found that CKD was an independent predictor for both device-oriented composite endpoint (DOCE) and BARC 3–5; however, ≥3 lesions treated or ≥3 stents implanted were the only independent predictors for DOCE, but not bleeding. On the contrary, in the present study, each component of complex PCI procedures was not associated with clinically relevant bleeding. Thereby, the high proportion of CKD of the “complex PCI” in the Bern PCI Registry is predisposed to clinical tendencies to bleeding events. Additionally, one explanation for these differences could lie in the use of P2Y_12_ inhibitors. Prior reports involved use of more potent antiplatelet agents such as ticagrelor and prasugrel which cause more bleeding events despite of lowering residual ischemic risk [7–9, 12], whereas the patients from our current study were limited to treated with clopidogrel due to the unavailability of other P2Y_12_ inhibitors except for clopidogrel in China during the study period. Moreover, another hypothetical explanation for this negative result of clinically relevant bleeding may reside in the bleeding risk of the study population. The mean PARIS bleeding risk score in our cohort was relatively lower than the Bern PCI Registry (3.7 ± 2.1 vs. 4.3 ± 2.5). Meanwhile, it was speculated that the data on comparisons of triple therapy (oral anticoagulant (OAC), aspirin, and P2Y_12_ inhibitor) versus double (OAC plus P2Y_12_ inhibitor) demonstrated that triple therapy significantly increased the risk of bleeding [26, 27], an important consideration given that up to 8.3% of patients treated with triple therapy (any DAPT and oral anticoagulant) at discharge in the Bern PCI Registry compared with 0.2% in our PCI registry.

Although the risks of ischemic events are greater than those of major bleeding in most patients undergoing PCI, the predicted long-term probabilities of ischemic (composite of cardiac death, MI, and definite/probable ST) and bleeding (moderate/severe bleeding) outcomes may share common risk factors and have a strong correlation [28]. Given the mutual and possibly competing role of high ischemic and bleeding risk features, we postulated that HBR may differentially affect the risk of adverse events after complex PCI compared with noncomplex PCI. Nevertheless, the relevance of complex PCI procedures on longitudinal outcomes in the setting of HBR remains less clear. In the present investigation, we found that the presence of HBR further amplified the underlying thrombotic risk of complex PCI because subjects with both abnormalities were at highest risk for ischemic events and those without complex PCI and absent HBR were at lower ischemic risk; however, the association between complex PCI and ischemic outcomes was similar in direction and magnitude among those with and without HBR, with no evidence of statistical interaction. In other words, HBR increased the risk of adverse ischemic events to a similar extent after complex PCI and noncomplex PCI. Similarly, no evidence of an interaction between complex PCI and HBR in regard to the risk of clinically relevant bleeding was observed, although the unadjusted rates of bleeding complications were numerically higher for HBR patients with vs. without complex PCI. These observations suggested that more intense and longer antiplatelet therapy may improve outcomes after complex PCI procedures. Nonetheless, the potential implications from our findings should all be considered as hypothesis-generating and require randomized trials for validation.

This study needs to be interpreted in the context of certain limitations. First, this study was a post hoc analysis of an observational, albeit large, prospective study that precluded causal inferences, and as such, it had to be considered as hypothesis-generating. Second, the patients who underwent complex PCI were not randomly assigned but were decided according to the operator's discretion. Although the major results were consistent after multivariable adjustment models, we did not correct for all possible and unmeasured confounders. Third, significant differences in the adjusted rates of clinical outcomes in HBR patients with complex PCI vs. noncomplex PCI were not observed, in contrast to that seen in the non-HBR cohort and the entire study population. This condition was likely due to the relatively modest sample size of HBR patients. No significant interactions were present between complex PCI and HBR versus non-HBR status for the risk of ischemic and bleeding events, indicating that the primary results of the study as related to the influence of complex PCI on clinical outcomes apply to HBR patients as well. Fourth, as all patients in our study only received clopidogrel as the P2Y_12_ inhibitor, the results may not be generalizable to those receiving more potent antiplatelet agents, such as ticagrelor or prasugrel. Despite these limitations, the current study had the advantages of inclusion of an all-comers population with minimal exclusion criteria, full-scale procedural complexity definitions, and a relatively long-term follow-up duration.

## 5. Conclusions

Patients having complex PCI procedures, compared to those having noncomplex PCI, were at a substantial higher risk of ischemic events, with no higher risk of clinically relevant bleeding, irrespective of HBR status. HBR further increased the risk of long-term adverse events after PCI of both complex PCI and noncomplex PCI to a comparable degree, whereas bleeding risk did not increase to the same extent as ischemic risk after complex PCI procedures within HBR group, suggesting that intensified antiplatelet therapy may be beneficial for patients with complex PCI.

## Figures and Tables

**Table 1:** Baseline characteristics according to PCI complexity.

	Complex PCI (*n* = 3651)	Noncomplex PCI (*n* = 6516)	*P* value
*Baseline characteristics*			
Age, years	58.62 ± 10.00	58.15 ± 10.39	0.026
Male	2897 (79.3)	4944 (75.9)	<0.001
Body mass index, kg/m^2^	26.05 ± 3.17	25.86 ± 3.19	0.003
Hypertension	2413 (66.1)	4128 (63.4)	0.006
Diabetes mellitus	1202 (32.9)	1840 (28.2)	<0.001
Dyslipidemia	2498 (68.4)	4339 (66.6)	0.059
Chronic kidney disease	158 (4.3)	246 (3.8)	0.171
Current smoker	2137 (58.5)	3677 (56.4)	0.040
Prior MI	825 (22.6)	1095 (16.8)	<0.001
Prior PCI	968 (26.5)	1453 (22.3)	<0.001
Prior CABG	182 (5.0)	221 (3.4)	<0.001
Prior cerebrovascular event	396 (10.8)	684 (10.5)	0.584
Prior PAD	108 (3.0)	159 (2.4)	0.117
LVEF, (%)	62.44 ± 7.40	63.07 ± 7.14	<0.001
ACS	2058 (56.4)	4036 (61.9)	<0.001
Hemoglobin, g/dL	14.29 ± 1.52	14.30 ± 1.54	0.878
Platelet count, 10^9^/L	204.01 ± 52.84	206.63 ± 56.71	0.022
White blood cell count, 10^9^/L	6.78 ± 1.64	6.72 ± 1.71	0.122
eGFR, ml/min/1.73 m^2^	94.68 ± 18.77	95.27 ± 18.35	0.125
PARIS thrombotic risk score	2.60 ± 1.73	2.46 ± 1.65	<0.001
PARIS bleeding risk score	3.72 ± 2.08	3.70 ± 2.08	0.600
Duration of DAPT, days	577.86 ± 209.19	563.29 ± 207.40	<0.001
*Procedural characteristics*			
Target vessel location			
Left main	268 (7.3)	0 (0.0)	<0.001
Left anterior descending artery	3036 (83.2)	6139 (94.2)	<0.001
Left circumflex artery	1283 (35.1)	525 (8.1)	<0.001
Right coronary artery	1402 (38.4)	473 (7.3)	<0.001
Bypass graft	10 (0.3)	7 (0.1)	0.049
In-stent restenosis target lesion	447 (12.2)	0 (0.0)	<0.001
Heavy calcified lesion (using rotablation)	52 (1.4)	0 (0.0)	<0.001
Thrombotic lesion	169 (4.6)	226 (3.5)	0.004
Type B2/C lesion	3335 (91.3)	4477 (68.7)	<0.001
SYNTAX score	15.0 (9.0–21.0)	8.0 (5.0–13.0)	<0.001
Total lesion length, mm	60.0 (42.0–76.0)	24.0 (16.0–35.0)	<0.001
Number of vessels treated			
1	1798 (49.2)	5888 (90.4)	<0.001
2	1626 (44.5)	628 (9.6)	<0.001
3	227 (6.2)	0 (0.0)	<0.001
Number of lesions stented	1.88 ± 0.83	1.16 ± 0.37	<0.001
Number of stents implanted	2.86 ± 1.13	1.39 ± 0.49	<0.001
Bifurcation treated with 2 stents	428 (11.7)	0 (0.0)	<0.001
Chronic total occlusion treated	836 (22.9)	0 (0.0)	<0.001
Total stent length, mm	64.0 (45.0–81.0)	28.0 (18.0–38.0)	<0.001
Total stent length>60 mm	2052 (56.2)	0 (0.0)	<0.001
Mean stent diameter, mm	2.90 ± 0.53	3.08 ± 0.56	<0.001
Glycoprotein IIb/IIIa use	801 (21.9)	848 (13.0)	<0.001
Type of DES implanted			0.503
Early-generation DES	388 (10.6)	665 (10.2)	
New-generation DES	3263 (89.4)	5851 (89.8)	
Radial approach	3274 (89.7)	5997 (92.0)	<0.001
Use of intravascular ultrasound	338 (9.3)	212 (3.3)	<0.001

Values are mean ± SD or median (interquartile range) for continuous variables, and *n* (%) for categorical variables. ACS = acute coronary syndrome (s); CAD = coronary artery disease; CABG = coronary artery bypass grafting; DAPT = dual antiplatelet therapy; DES = drug-eluting stents; EF = ejection fraction; LVEF = left ventricular ejection fraction; MI = myocardial infarction; NSTEMI = non-ST-segment elevation myocardial infarction; PAD = peripheral artery disease; PCI = percutaneous coronary intervention; PARIS= Patterns of Nonadherence to Antiplatelet Regimen in Stented Patients; STEMI = ST-segment elevation myocardial infarction.

**Table 2:** Clinical outcomes according to PCI complexity.

	Complex PCI (*n* = 3651)	Noncomplex PCI (*n* = 6516)	Unadjusted	*P* value	MV adjusted^*∗*^	*P* value
			HR (95% CI)		HR (95% CI)	
MACE^a^	280 (7.7%)	290 (4.5%)	1.77 (1.50–2.08)	<0.001	1.63 (1.38–1.92)	<0.001
Death	54 (1.5%)	80 (1.2%)	1.20 (0.85–1.69)	0.307	1.22 (0.85–1.75)	0.275
Cardiac death	33 (0.9%)	39 (0.6%)	1.50 (0.94–2.38)	0.088	1.50 (0.92–2.44)	0.108
MI	109 (3.0%)	87 (1.3%)	2.25 (1.70–3.00)	<0.001	2.16 (1.62–2.87)	<0.001
Target vessel MI	52 (1.4%)	37 (0.6%)	2.51 (1.64–3.82)	<0.001	2.50 (1.59–3.77)	<0.001
Definite or probable ST	44 (1.2%)	27 (0.4%)	2.91 (1.80–4.69)	<0.001	2.71 (1.66–4.41)	<0.001
Any revascularization	400 (11.0%)	487 (7.5%)	1.50 (1.32–1.72)	<0.001	1.38 (1.21–1.58)	<0.001
TVR	234 (6.4%)	249 (2.9%)	1.72 (1.44–2.05)	<0.001	1.56 (1.30–1.87)	<0.001
TLR	182 (5.0%)	190 (2.9%)	1.75 (1.43–2.14)	<0.001	1.59 (1.29–1.95)	<0.001
Stroke	72 (2.0%)	94 (1.4%)	1.35 (0.99–1.83)	0.058	1.36 (1.00–1.86)	0.053
Any bleeding	229 (6.3%)	467 (7.2%)	0.87 (0.74–1.01)	0.074	0.91 (0.77–1.07)	0.245
Clinically relevant bleeding^b^	87 (2.4%)	191 (2.9%)	0.80 (0.62–1.03)	0.088	0.86 (0.66–1.11)	0.238

Values are number of events (%) unless otherwise indicated. ^*∗*^The following covariates have been included in the Cox regression multivariable model: age, sex, current smoking, body mass index, hypertension, diabetes mellitus, chronic kidney disease, left ventricular ejection fraction, prior MI, prior revascularization (percutaneous coronary intervention and/or coronary artery bypass graft), acute coronary syndrome, mean stent diameter, hemoglobin, platelet count, type of DES implanted, and DAPT duration (as a time-adjusted covariate). BARC = Bleeding Academic Research Consortium; CI = confidence interval; HR = hazard ratio; MACE = major adverse cardiac events; ST = stent thrombosis; TVR = target vessel revascularization; TLR = target lesion revascularization; other abbreviations as in Table 1^a^ MACE was defined as the composite of cardiac death, myocardial infarction, definite/probable stent thrombosis, or target lesion revascularization. ^b^ Clinically relevant bleeding was defined as BARC type 2, 3, or 5.

**Table 3:** HRs for adverse events associated with complex PCI stratified by PARIS bleeding risk score.

	PARIS bleeding risk score<8 (non-HBR)			PARIS bleeding risk score≥8 (HBR)			
	Complex PCI (*n* = 3479)	Noncomplex PCI (*n* = 6218)	Adjusted HR (95% CI) ^*∗*^	Complex PCI (*n* = 172)	Noncomplex PCI (*n* = 298)	Adjusted HR (95% CI) ^*∗*^	*P* value for interaction
MACE^a^	265 (7.6%)	270 (4.3%)	1.66 (1.40–1.97)	15 (8.7%)	20 (6.7%)	1.13 (0.57–2.25)	0.388
Death	45 (1.3%)	64 (1.0%)	1.30 (0.87–1.93)	9 (5.2%)	15 (5.0%)	0.83 (0.33–2.11)	0.496
Cardiac death	26 (0.7%)	29 (0.5%)	1.63 (0.94–2.84)	7 (4.1%)	9 (3.0%)	1.15 (0.37–3.58)	0.519
MI	101 (2.9%)	80 (1.3%)	2.19 (1.62–2.95)	8 (4.7%)	7 (2.3%)	1.57 (0.51–4.83)	0.720
Definite/probable ST	39 (1.3%)	21 (0.5%)	3.14 (1.83–5.39)	5 (2.9%)	6 (2.0%)	1.42 (0.38–5.29)	0.146
TLR	175 (5.0%)	182 (2.9%)	1.59 (1.29–1.97)	7 (4.1%)	8 (2.7%)	1.52 (0.51–4.46)	0.886
Clinically relevant bleeding^b^	77 (2.2%)	178 (2.9%)	0.82 (0.62–1.07)	10 (5.8%)	13 (4.4%)	1.22 (0.52–2.85)	0.279

Values are number of events (%) unless otherwise indicated.  ^*∗*^The following covariates have been included in the Cox regression multivariable model: age, sex, current smoking, body mass index, hypertension, diabetes mellitus, chronic kidney disease, left ventricular ejection fraction, prior MI, prior revascularization (percutaneous coronary intervention and/or coronary artery bypass graft), acute coronary syndrome, mean stent diameter, hemoglobin, platelet count, type of DES implanted, and DAPT duration (as a time-adjusted covariate). Abbreviations as in Table 1 and Table 2^a^ MACE was defined as the composite of cardiac death, myocardial infarction, definite/probable stent thrombosis, or target lesion revascularization. ^b^ Clinically relevant bleeding was defined as BARC type 2, 3, or 5.

## Data Availability

The data used to support the findings of this study are available from the corresponding author upon reasonable request.

## Supplementary Materials

Supplementary Materials It includes the supplementary methods, Table, and Figure that need to show online. Supplementary Table 1: overlap between components of complex PCI. Supplementary Table 2: ischemic and bleeding events according to the number of complex PCI features and complex PCI score. Supplementary Table 3: effect of each high-risk procedural subset on MACE and clinically relevant bleeding. Supplementary Table 4: event rates in subjects with versus without HBR after coronary stenting. Supplementary Table 5: HRs for adverse events associated with complex PCI stratified by PARIS bleeding risk score category (low, intermediate, or high). Supplementary Table 6: HRs for adverse events associated with complex PCI stratified by PRECISE-DAPT score. Supplementary Figure 1: prevalence and overlap of complex PCI components in the overall population. PCI = percutaneous coronary intervention.
