# Supplementary material for: Percutaneous Coronary Intervention Complexity and Risk of Adverse Events in relation to High Bleeding Risk among Patients Receiving Drug-Eluting Stents: Insights from a Large Single-Center Cohort Study
Source: J Interv Cardiol. 2020 Apr 25;2020:2985435. doi: 10.1155/2020/2985435 (PMC7196972; doi:10.1155/2020/2985435)
Supplement: Supplementary Materials — It includes the supplementary methods, Table, and Figure that need to show online. Supplementary Table 1: overlap between components of complex PCI. Supplementary Table 2: ischemic and bleeding events according to the number of complex PCI features and complex PCI score. Supplementary Table 3: effect of each high-risk procedural subset on MACE and clinically relevant bleeding. Supplementary Table 4: event rates in subjects with versus without HBR after coronary stenting. Supplementary Table 5: HRs for adverse events associated with complex PCI stratified by PARIS bleeding risk score category (low, intermediate, or high). Supplementary Table 6: HRs for adverse events associated with complex PCI stratified by PRECISE-DAPT score. Supplementary Figure 1: prevalence and overlap of complex PCI components in the overall population. PCI = percutaneous coronary intervention. [file 2985435.f1.docx]

**SUPPLEMENTAL MATERIAL**

Title: Percutaneous coronary intervention complexity and risk of adverse events in relation to high bleeding risk among patients receiving drug-eluting stents: Insights from a large single-center cohort study

**Supplemental Methods**

**Clinical and laboratory analysis**

Body mass index (BMI) was calculated as weight per height squared (kg/m^2^). The subjects were reminded of keeping an overnight fasting with 12 hours before the investigation. Venous blood specimens were designed to be obtained for assessing total cholesterol (TC), triglyceride (TG), low-density lipoprotein cholesterol (LDL-C), high-density lipoprotein cholesterol (HDL-C), fasting plasma glucose (FPG) and serum creatinine by automatic Biochemistry analyzer (Hitachi 7150, Tokyo, Japan). The definition of hypertension was established as blood pressure (BP) level of at least 140/90 mmHg, individuals who were on antihypertensive medications or a prior diagnosis of hypertension. Diabetes was recognized in patients with a fasting glucose ≥7.0 mmol/L, or the 2-h plasma glucose of the oral glucose tolerance test ≥11.0 mmol/L, or current use of hypoglycemic drugs or insulin. Chronic kidney disease (CKD) is defined as glomerular filtration rate <60 ml/min/1.73 m^2^ using the CKD-Epidemiology collaboration definition [1].

**Procedures**

The PCI procedure and best available medical therapy were performed in accordance with the current procedural guidelines [2, 3]. All patients received a loading dose of aspirin (300 mg) and clopidogrel 300 to 600 mg before PCI unless they had previously received these antiplatelet medications. Unfractionated heparin at a dose of 100 IU/kg was administered during the procedure to achieve an activated clotting time of 250 to 300s. The access site, revascularization treatment strategy, periprocedural use of glycoprotein IIb/IIIa receptor inhibitors, use of intravascular ultrasound, and selection of DES was left to the discretion of the operator. After the index procedure, aspirin at a dose of 100 mg once daily was continued indefinitely, and clopidogrel (75 mg daily) was recommended for at least one year. Optimal medications therapy, including β-blocker, renin-angiotensin system blockade, nitrates, calcium channel blocker, and statins were also recommended to all patients at the discretion of the responsible clinicians.

**Definitions and clinical outcomes**

All deaths were considered cardiac in origin unless a definite noncardiac cause could be documented. According to the third universal definition of MI, MI was defined as an elevation of creatine kinase-myocardial band or troponin level greater than the upper limit of normal with the presence of ischemic symptoms, new electrocardiographic abnormalities suggestive of ischemia [4]. Stent thrombosis was categorized according to the definitions provided by the Academic Research Consortium [5]. TLR was defined as any repeat PCI or coronary bypass graft surgery (CABG) due to a stenosis or occlusion within the stent or within the 5 mm borders adjacent to the stent. TVR was defined as any revascularization within the entire major coronary vessels proximal or distal to a target lesion by repeat PCI or CABG. Stroke, as detected by the occurrence of a new neurological deficit, was confirmed by a neurologist and imaging. Bleeding events were categorized on the basis of the BARC classifications. We classified BARC types 2, 3, or 5 bleeding as clinically relevant bleeding for the present analysis.

**References:**

[1] Levey AS, Stevens LA, Schmid CH, Zhang YL, Castro AF, 3rd, Feldman HI, et al. A new equation to estimate glomerular filtration rate. Annals of internal medicine. 2009;150:604-12.

[2] Neumann FJ, Sousa-Uva M, Ahlsson A, Alfonso F, Banning AP, Benedetto U, et al. 2018 ESC/EACTS Guidelines on myocardial revascularization. European heart journal. 2019;40:87-165.

[3] Levine GN, Bates ER, Blankenship JC, Bailey SR, Bittl JA, Cercek B, et al. 2011 ACCF/AHA/SCAI Guideline for Percutaneous Coronary Intervention. A report of the American College of Cardiology Foundation/American Heart Association Task Force on Practice Guidelines and the Society for Cardiovascular Angiography and Interventions. Journal of the American College of Cardiology. 2011;58:e44-122.

[4] Thygesen K, Alpert JS, Jaffe AS, Simoons ML, Chaitman BR, White HD, et al. Third universal definition of myocardial infarction. Journal of the American College of Cardiology. 2012;60:1581-98.

[5] Cutlip DE, Windecker S, Mehran R, Boam A, Cohen DJ, van Es GA, et al. Clinical end points in coronary stent trials: a case for standardized definitions. Circulation. 2007;115:2344-51.

Supplementary Table 1: Overlap between components of complex PCI.

|  | 3 vessels treated | ≥3 stents implanted | ≥3 lesions treated | Bifurcation with 2 stents | >60 mm total stent length | Chronic total occlusion | Unprotected left main | In-stent restenosis target lesion | Severely calcified lesion (requiring a rotablator system) |
| --- | --- | --- | --- | --- | --- | --- | --- | --- | --- |
| 3 vessels treated (n=227) |  | 209 (92.1%) | 222 (97.8%) | 25 (11.0%) | 194 (85.5%) | 49 (21.6%) | 42 (18.5%) | 16 (7.0%) | 0 (0%) |
| ≥3 stents implanted (n=2385) | 209 (8.8%) |  | 598 (25.1%) | 262 (11.0%) | 1817 (76.2%) | 394 (16.5%) | 178 (7.5%) | 93 (3.9%) | 23 (1.0%) |
| ≥3 lesions treated (n=734) | 222 (30.2%) | 598 (81.5%) |  | 91 (12.4%) | 500 (68.1%) | 137 (18.7%) | 98 (13.4%) | 52 (7.1%) | 6 (0.8%) |
| Bifurcation with 2 stents (n=428) | 25 (5.8%) | 262 (61.2%) | 91 (21.3%) |  | 163 (38.1%) | 39 (9.1%) | 96 (22.4%) | 15 (3.5%) | 1 (0.2%) |
| >60 mm total stent length (n=2052) | 194 (9.5%) | 1817 (88.5%) | 500 (24.4%) | 163 (7.9%) |  | 382 (18.6%) | 137 (6.7%) | 68 (3.3%) | 19 (0.9%) |
| Chronic total occlusion (n=836) | 49 (5.9%) | 394 (47.1%) | 137 (16.4%) | 39 (4.7%) | 382 (45.7%) |  | 31 (3.7%) | 56 (6.7%) | 6 (0.7%) |
| Unprotected left main (n=268) | 42 (15.7%) | 178 (66.4%) | 98 (36.6%) | 96 (35.8%) | 137 (51.1%) | 31 (11.6%) |  | 11 (4.1%) | 1 (0.4%) |
| In-stent restenosis target lesion (n=447) | 16 (3.6%) | 93 (20.8%) | 52 (11.6%) | 15 (3.4%) | 68 (15.2%) | 56 (12.5%) | 11 (2.5%) |  | 1 (0.2%) |
| Severely calcified lesion (requiring a rotablator system) (n=52) | 0 (0%) | 23 (44.2%) | 6 (11.5%) | 1 (1.9%) | 19 (36.5%) | 6 (11.5%) | 11 (1.9%) | 1 (1.9%) |  |

Values are n (%).

Supplementary Table 2: Ischemic and bleeding events according to the number of complex PCI features and complex PCI score

|  | No. of Events (%) | Unadjusted HR (95%CI) | P value | Adjusted HR (95% CI)† | P value |
| --- | --- | --- | --- | --- | --- |
| **MACE^a^** |  |  |  |  |  |
| Complex PCI score* | 570 (5.6) | 1.19 (1.13-1.27) | <0.001 | 1.16 (1.09-1.23) | <0.001 |
| Number of complexity characteristics |  |  |  |  |  |
| 0 | 290 (4.5) | 1.00 (reference) |  | 1.00 (reference) |  |
| 1-2 | 197 (7.4) | 1.71 (1.43-2.05) | <0.001 | 1.58 (1.32-1.90) | <0.001 |
| ≥3 | 83 (8.3) | 1.92 (1.51-2.45) | <0.001 | 1.75 (1.37-2.24) | <0.001 |
| P for trend |  |  | <0.001 |  | <0.001 |
| **Cardiac death** |  |  |  |  |  |
| Complex PCI score* | 72 (0.7) | 1.16 (0.98-1.38) | <0.001 | 1.20 (1.01-1.42) | 0.038 |
| Number of complexity characteristics |  |  |  |  |  |
| 0 | 39 (0.6) | 1.00 (reference) |  | 1.00 (reference) |  |
| 1-2 | 22 (0.8) | 1.38 (0.82-2.33) | 0.229 | 1.27 (0.72-2.22) | 0.408 |
| ≥3 | 11 (1.1) | 1.81 (0.93-3.54) | 0.082 | 2.18 (1.09-4.36) | 0.028 |
| P for trend |  |  | 0.058 |  | 0.037 |
| **MI** |  |  |  |  |  |
| Complex PCI score* | 196 (1.9) | 1.41 (1.29-1.54) | <0.001 | 1.40 (1.28-1.53) | <0.001 |
| Number of complexity characteristics |  |  |  |  |  |
| 0 | 87 (1.3) | 1.00 (reference) |  | 1.00 (reference) |  |
| 1-2 | 58 (2.2) | 1.64 (1.18-2.29) | 0.004 | 1.56 (1.11-2.18) | 0.010 |
| ≥3 | 51 (5.1) | 3.88 (2.74-5.48) | <0.001 | 3.84 (2.70-5.46) | <0.001 |
| P for trend |  |  | <0.001 |  | <0.001 |
| **Definite/probable ST** |  |  |  |  |  |
| Complex PCI score* | 71 (0.7) | 1.54 (1.35-1.77) | <0.001 | 1.53 (1.33-1.76) | <0.001 |
| Number of complexity characteristics |  |  |  |  |  |
| 0 | 27 (0.4) | 1.00 (reference) |  | 1.00 (reference) |  |
| 1-2 | 23 (0.9) | 2.09 (1.20-3.64) | 0.009 | 1.94 (1.10-3.41) | 0.021 |
| ≥3 | 21 (2.1) | 5.09 (2.88-9.00) | <0.001 | 4.78 (2.67-8.55) | <0.001 |
| P for trend |  |  | <0.001 |  | <0.001 |
| **TLR** |  |  |  |  |  |
| Complex PCI score* | 372 (3.7) | 1.14 (1.05-1.23) | 0.001 | 1.10 (1.01-1.18) | 0.023 |
| Number of complexity characteristics |  |  |  |  |  |
| 0 | 190 (2.9) | 1.00 (reference) |  | 1.00 (reference) |  |
| 1-2 | 142 (5.3) | 1.89 (1.52-2.34) | <0.001 | 1.74 (1.39-2.17) | <0.001 |
| ≥3 | 40 (4.0) | 1.39 (0.99-1.96) | 0.057 | 1.21 (0.86-1.70) | 0.285 |
| P for trend |  |  | <0.001 |  | 0.002 |
| **Clinically relevant bleeding^b^** |  |  |  |  |  |
| Complex PCI score* | 278 (2.7) | 0.89 (0.80-1.00) | 0.044 | 0.91 (0.82-1.02) | 0.107 |
| Number of complexity characteristics |  |  |  |  |  |
| 0 | 191 (2.9) | 1.00 (reference) |  | 1.00 (reference) |  |
| 1-2 | 65 (2.4) | 0.83 (0.62-1.10) | 0.184 | 0.88 (0.66-1.17) | 0.364 |
| ≥3 | 22 (2.2) | 0.74 (0.47-1.15) | 0.176 | 0.80 (0.51-1.25) | 0.329 |
| P for trend |  |  | 0.083 |  | 0.223 |

*Complex PCI score represent the discrete number of elements qualifying for PCI complexity (e.g. presence of three lesions treated and bifurcation with 2 stents = 2 points). †The following covariates have been included in the Cox regression multivariable model: age, sex, current smoking, body mass index, hypertension, diabetes mellitus, chronic kidney disease, left ventricular ejection fraction, prior MI, prior revascularization (percutaneous coronary intervention and/or coronary artery bypass graft), acute coronary syndrome, mean stent diameter, hemoglobin, platelet count, type of DES implanted, and DAPT duration (as a time-adjusted covariate).

BARC = Bleeding Academic Research Consortium; CI = confidence interval; HR = hazard ratio; MACE = major adverse cardiac events; PCI = percutaneous coronary intervention; ST = stent thrombosis; TVR = target vessel revascularization; TLR = target lesion revascularization

^a^ MACE was defined as the composite of cardiac death, myocardial infarction, definite/probable stent thrombosis, or target lesion revascularization.

^b^ Clinically relevant bleeding was defined as BARC type 2, 3, or 5.

Supplementary Table 3: Effect of each high-risk procedural subsets on MACE and clinically relevant bleeding

|  | MACE | | Clinically relevant bleeding | |
| --- | --- | --- | --- | --- |
|  | Adjusted HR (95% CI)* | P value | Adjusted HR (95% CI)* | P value |
| 3 vessels treated | 0.89 (0.51-1.55) | 0.687 | 0.50 (0.16-1.56) | 0.230 |
| ≥3 stents implanted | 1.48 (1.24-1.77) | <0.001 | 0.81 (0.60-1.09) | 0.160 |
| ≥3 lesions treated | 1.30 (0.98-1.72) | 0.066 | 0.85 (0.51-1.42) | 0.539 |
| Bifurcation with 2 stents | 1.46 (1.03-2.08) | 0.034 | 0.98 (0.54-1.80) | 0.956 |
| >60mm total stent length | 1.40 (1.16-1.69) | <0.001 | 0.92 (0.67-1.24) | 0.572 |
| Chronic total occlusion | 1.27 (0.98-1.66) | 0.076 | 0.74 (0.44-1.24) | 0.251 |
| Unprotected left main | 1.47 (0.97-2.24) | 0.072 | 0.90 (0.42-1.90) | 0.772 |
| In-stent restenosis target lesion | 1.80 (1.31-2.46) | <0.001 | 0.40 (0.16-0.99) | 0.047 |
| Severely calcified lesion | 2.26 (1.06-4.79) | 0.034 | 1.19 (0.30-4.84) | 0.804 |

* The following covariates have been included in the Cox regression multivariable model: age, sex, current smoking, body mass index, hypertension, diabetes mellitus, chronic kidney disease, left ventricular ejection fraction, prior MI, prior revascularization (percutaneous coronary intervention and/or coronary artery bypass graft), acute coronary syndrome, mean stent diameter, hemoglobin, platelet count, type of DES implanted, and DAPT duration (as a time-adjusted covariate). Abbreviations as in Supplementary Table 2.

Supplementary Table 4: Event rates in subjects with versus without HBR after coronary stenting

|  | HBR (n=470) | Non-HBR (n=9697) | HR (95% CI) | P value |
| --- | --- | --- | --- | --- |
| Death | 24 (5.1%) | 109 (1.1%) | 4.64 (2.98-7.21) | <0.001 |
| Cardiac death | 16 (3.4%) | 56 (0.6%) | 6.06 (3.48-10.57) | <0.001 |
| MI | 15 (3.2%) | 181 (1.9%) | 1.74 (1.03-2.94) | 0.040 |
| TV-MI | 12 (2.6%) | 77 (0.8%) | 3.31 (1.80-6.08) | <0.001 |
| Definite/probable ST | 11 (2.3%) | 60 (0.6%) | 3.88 (2.04-7.38) | <0.001 |
| Any revascularization | 34 (7.2%) | 853 (8.8%) | 0.85 (0.60-1.19) | 0.335 |
| TLR | 15 (3.2%) | 357 (3.7%) | 0.89 (0.53-1.49) | 0.659 |
| Stroke | 15 (3.2%) | 150 (1.6%) | 2.13 (1.25-3.62) | 0.005 |
| Clinically relevant bleeding | 23 (4.9%) | 255 (2.6%) | 1.94 (1.26-2.97) | 0.002 |

HBR = high bleeding risk; other abbreviations as in Supplementary Table 2.

Supplementary Table 5: HRs for adverse events associated with complex PCI stratified by PARIS bleeding risk score category (low, intermediate, or high)

|  | Low Bleeding Risk (0-3) | | | Intermediate Bleeding Risk (4-7) | | | High Bleeding Risk (≥8) | | |  |
| --- | --- | --- | --- | --- | --- | --- | --- | --- | --- | --- |
|  | Complex PCI (n=1849) | Noncomplex PCI (n=3329) | Adjusted HR (95% CI)* | Complex PCI (n=1630) | Noncomplex PCI (n=2889) | Adjusted HR (95% CI)* | Complex PCI (n=172) | Noncomplex PCI (n=298) | Adjusted HR (95% CI)* | P value for interaction |
| MACE | 141 (7.6) | 145 (4.4) | 1.61 (1.27-2.05) | 124 (7.6) | 125 (4.3) | 1.70 (1.32-2.19) | 15 (8.7) | 20 (6.7) | 1.13 (0.57-2.25) | 0.680 |
| Death | 23 (1.2) | 20 (0.6) | 2.40 (1.25-4.61) | 22 (1.3) | 45 (1.6) | 0.90 (0.53-1.52) | 9 (5.2) | 15 (5.0) | 0.83 (0.33-2.11) | 0.103 |
| Cardiac death | 13 (0.7) | 14 (0.4) | 1.73 (0.76-4.00) | 13 (0.8) | 16 (0.6) | 1.66 (0.76-3.64) | 7 (4.1) | 9 (3.0) | 1.15 (0.37-3.58) | 0.830 |
| MI | 52 (2.8) | 37 (1.1) | 2.42 (1.57-3.72) | 49 (3.0) | 43 (1.5) | 2.00 (1.32-3.04) | 8 (4.7) | 7 (2.3) | 1.57 (0.51-4.83) | 0.686 |
| Definite/probable ST | 21 (1.1) | 12 (0.4) | 2.89 (1.40-5.97) | 18 (1.1) | 9 (0.3) | 3.78 (1.67-8.58) | 5 (2.9) | 6 (2.0) | 1.42 (0.38-5.29) | 0.356 |
| TLR | 99 (5.4) | 107 (3.2) | 1.52 (1.15-2.02) | 76 (4.7) | 75 (2.6) | 1.69 (1.22-2.34) | 7 (4.1) | 8 (2.7) | 1.52 (0.51-4.46) | 0.852 |
| Clinically relevant bleeding | 37 (2.0) | 74 (2.2) | 0.96 (0.64-1.44) | 40 (2.5) | 104 (3.6) | 0.72 (0.50-1.04) | 10 (5.8) | 13 (4.4) | 1.22 (0.52-2.85) | 0.301 |

* The following covariates have been included in the Cox regression multivariable model: age, sex, current smoking, body mass index, hypertension, diabetes mellitus, chronic kidney disease, left ventricular ejection fraction, prior MI, prior revascularization (percutaneous coronary intervention and/or coronary artery bypass graft), acute coronary syndrome, mean stent diameter, hemoglobin, platelet count, type of DES implanted, and DAPT duration (as a time-adjusted covariate).

PARIS = Patterns of Non-Adherence to Anti-Platelet Regimen in Stented Patients; other abbreviations as in Supplementary Table 2.

Supplementary Table 6: HRs for adverse events associated with complex PCI stratified by PRECISE-DAPT score

|  | PRECISE-DAPT score<25 (non-HBR) | | | PRECISE-DAPT score≥25 (HBR) | | |  |
| --- | --- | --- | --- | --- | --- | --- | --- |
|  | Complex PCI (n=3414) | Noncomplex PCI (n=6071) | Adjusted HR (95% CI)* | Complex PCI (n=237) | Noncomplex PCI (n=445) | Adjusted HR (95% CI)* | P value for interaction |
| MACE^a^ | 248 (7.3%) | 265 (4.4%) | 1.56 (1.31-1.86) | 32 (13.5%) | 25 (5.6%) | 2.38 (1.38-4.09) | 0.118 |
| Death | 41 (1.2%) | 64 (1.1%) | 1.08 (0.72-1.63) | 13 (5.5%) | 16 (3.6%) | 1.82 (0.80-4.18) | 0.108 |
| Cardiac death | 33 (0.7%) | 30 (0.5%) | 1.25 (0.70-2.22) | 10 (4.2%) | 9 (2.0%) | 2.51 (0.86-7.35) | 0.121 |
| MI | 87 (2.5%) | 79 (1.3%) | 1.87 (1.37-2.55) | 22 (9.3%) | 8 (1.8%) | 5.26 (2.28-12.13) | 0.031 |
| Definite/probable ST | 33 (1.0%) | 22 (0.4%) | 2.46 (1.42-4.26) | 11 (4.6%) | 5 (1.1%) | 5.00 (1.60-15.56) | 0.571 |
| TLR | 172 (5.0%) | 180 (3.0%) | 1.57 (1.27-1.95) | 10 (4.2%) | 10 (2.2%) | 1.80 (0.71-4.51) | 0.585 |
| Clinically relevant bleeding^b^ | 74 (2.2%) | 168 (2.8%) | 0.82 (0.62-1.08) | 13 (5.5%) | 23 (5.2%) | 1.17 (0.58-2.36) | 0.463 |

Values are number of events (%) unless otherwise indicated. *The following covariates have been included in the Cox regression multivariable model: age, sex, current smoking, body mass index, hypertension, diabetes mellitus, chronic kidney disease, left ventricular ejection fraction, prior MI, prior revascularization (percutaneous coronary intervention and/or coronary artery bypass graft), acute coronary syndrome, mean stent diameter, hemoglobin, platelet count, type of DES implanted, and DAPT duration (as a time-adjusted covariate).

^a^ MACE was defined as the composite of cardiac death, myocardial infarction, definite/probable stent thrombosis, or target lesion revascularization.

^b^ Clinically relevant bleeding was defined as BARC type 2, 3, or 5.


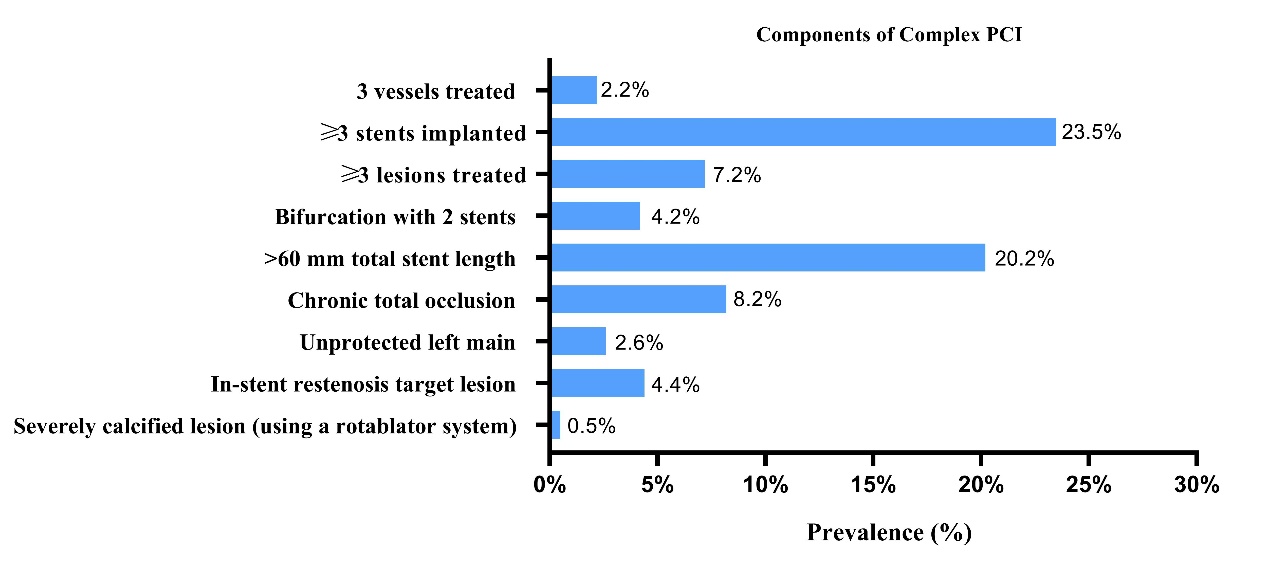


Supplementary Figure 1. Prevalence and overlap of complex PCI components in the overall population. PCI = percutaneous coronary intervention.
